# Norepinephrine Versus Dopamine as a First-Line Vasopressor in Dogs with Hypotension: A Pilot Study

**DOI:** 10.3390/vetsci12090832

**Published:** 2025-08-29

**Authors:** Bridget Lyons, Rebecka Hess, Deborah C. Silverstein

**Affiliations:** 1 Cornell University Veterinary Specialists, Stamford, CT 06902, USA; blyons@cuvs.org; 2 Department of Clinical Sciences and Advanced Medicine, University of Pennsylvania School of Veterinary Medicine, Philadelphia, PA 19104, USA

**Keywords:** hypotension, vasodilation, catecholamine, vasopressor, shock index

## Abstract

Norepinephrine (NE) and dopamine (DA) are vasopressors used to treat vasodilatory shock for decades, and norepinephrine is considered the preferred first-line vasopressor in human patients. However, there is a dearth of evidence to support specific treatment recommendations for the management of hypotensive, non-anesthetized, fluid-replete dogs. The objective of this study was to compare the effects of NE and DA on systolic blood pressure (SBP), heart rate, and shock index (SI) when used as first-line vasopressors for the treatment of vasodilatory shock in dogs. Twenty-four client-owned canine patients of similar age, sex, and weight with hypotension necessitating vasopressor therapy were randomized to receive NE or DA; attending clinicians were blinded. Twenty-two dogs were included in the final analysis (10 in the NE group and 12 in the DA group). Seventy-seven percent of all dogs achieved normotension. In both groups, SBP increased significantly compared to baseline (*p* = 0.0004 in the NE group and *p* = 0.006 in the DA group). The SI also decreased in both groups compared to baseline values (*p* = 0.01 in the NE group and *p* = 0.01 in the DA group). The heart rate in the NE group was higher than in the DA group at timepoints 6–10 (*p* = 0.023). Both NE and DA cause an increase in blood pressure and a decrease in SI in dogs with vasodilatory hypotension. Further investigation is warranted to determine if there are differences between NE and DA or the requirement for a second vasopressor, occurrence of arrhythmias, length of stay, and survival.

## Simple Summary

Diseases that cause severe inflammation can result in a decrease in blood pressure. If not treated appropriately, inadequate blood delivery to the major organs of the body can cause damage or failure. In addition to treating the underlying cause of severe inflammation, medical therapy to increase the low blood pressure is often administered. However, the most effective choice of vasopressor drugs to increase blood pressure in dogs is unknown. This study compared two commonly used medical treatments for low blood pressure, norepinephrine and dopamine, and evaluated their effectiveness and adverse effects. Both medications evaluated restored normal blood pressure in 77.3% of dogs with minimal adverse effects noted. This suggests that either drug is an appropriate first choice for the management of low blood pressure in dogs, but further research is needed.

## 1. Introduction

In contrast to other forms of shock that result in compensatory peripheral vasoconstriction, vasodilatory shock occurs due to a failure of vascular smooth muscle to constrict [1]. Sepsis is the most common cause of vasodilatory shock; however, it can also be caused by anaphylaxis, heat stroke, neurogenic shock, non-infectious causes of systemic inflammation, general anesthesia, and toxicoses from substances such as carbon monoxide, minoxidil, and calcium channel blockers [1,2,3]. One of the hallmarks of vasodilatory shock is vasoplegia, resulting in reduced systemic vascular resistance and arterial hypotension [3]. The administration of vasoactive medications that increase vasomotor tone is an essential component of therapy, as persistent vasodilatory shock results in decreased perfusion pressures and oxygen delivery, multiorgan failure, and death [3,4]. In human patients, delays in vasopressor initiation and restoration of adequate perfusion are associated with increased risk of organ failure and mortality [3,5].

Norepinephrine (NE) and dopamine (DA) are vasoactive medications that have been used to treat vasodilatory shock in humans and animals for decades. Norepinephrine acts on both alpha and beta-adrenergic receptors, although its actions on alpha-adrenergic receptors predominate, causing primarily vasoconstriction with minimal change in heart rate or contractility [6]. Dopamine has dose-dependent effects and acts primarily on DA receptors at low doses (1–3 mcg/kg/min), beta-adrenergic receptors at moderate doses (4–10 mcg/kg/min), mixed alpha and beta-adrenergic receptors at high doses (10–15 mcg/kg/min), and primarily alpha-adrenergic receptors at very high doses (>15 mcg/kg/min) [6,7]. The optimal first-line vasoactive agent for the treatment of vasodilatory shock has been unclear in veterinary medicine for many years. An experimental study in a canine model of sepsis using peritoneally implanted fibrin clots infected with *Escherichia coli* showed a differing response to NE (0.8–4 mcg/kg/min) and DA (5–20 mcg/kg/min) when compared with control dogs [8]. In this model, NE infusion resulted in increased mean arterial pressure (MAP) and decreased stroke volume and left ventricular ejection fraction compared to healthy controls, whereas the ability of DA to increase MAP was decreased compared to control dogs, but its ability to increase stroke volume and ejection fraction was maintained [8].

In contrast to the apparent beneficial and safe effects of dopamine in dogs, a comparison of NE (0.02–0.19 mcg/kg/min) and DA (2–20 mcg/kg/min) in human patients with undifferentiated shock found an increased risk of arrhythmias in the DA group, and an increase in mortality in patients with cardiogenic shock [9]. More recent evidence in humans with septic shock suggests that NE is associated with a decreased risk of arrhythmias and may be associated with decreased mortality compared to DA [10,11,12,13,14,15,16]. This has resulted in the current Surviving Sepsis Campaign’s recommendation to administer NE as the first-choice vasopressor for the treatment of sepsis-induced hypotension, with a further recommendation to administer vasopressin to patients with hypotension that is refractory to NE [17]. This recommendation has not been critically evaluated in dogs with vasodilatory shock, and data from the human literature may not be applicable to dogs as species-specific differences in sepsis-induced cytokine response, organ dysfunction, adrenergic receptors, response to vasoactive agents, and adverse effects may exist [18,19,20].

The primary objective of this pilot study was to compare the effects of NE and DA on systolic blood pressure, heart rate, and shock index when used as the first-line vasopressor in dogs diagnosed with vasodilatory shock. Secondary objectives included comparing length of stay, need for a second vasopressor, occurrence of arrhythmias, and mortality in dogs treated with NE or DA. It was hypothesized that both NE and DA would cause an increase in blood pressure in dogs with vasodilatory shock, and there would be no difference in the effectiveness or safety of the two drugs.

## 2. Materials and Methods

Dogs that fulfilled at least 2 out of the 4 systemic inflammatory response (SIRS) criteria with documented hypotension refractory to fluid administration that required vasopressor therapy by the attending clinician were eligible for inclusion. The previously defined SIRS criteria include the following: (rectal temperature < 37.8 °C (100.0 °F) or >39.4 °C (103.0 °F), heart rate > 120 bpm, respiratory rate > 40, white blood cell count within 24 h showing <6000/μL or >16,000/μL or >3% immature neutrophils). Hypotension was defined as a systolic blood pressure (SBP) ≤ 90 mmHg or a mean arterial blood pressure (MAP) ≤ 70 mmHg despite a minimum of a 15 mL/kg bolus of isotonic crystalloids or a 5 mL/kg bolus of a synthetic colloid. Patients less than six months of age were excluded due to variability in body temperature and heart rate in pediatric dogs compared to adults [21].

A randomization sequence was generated using a free online software program, https://www.randomizer.org/ (accessed on 2 October 2012), and the results were placed into sealed envelopes. Once enrolled, the veterinary nurse caring for the patient opened an envelope containing the letter A or B to represent whether the patient would receive NE (A) or DA (B) as a first-line vasopressor and formulated the constant rate infusion (CRI) as indicated using standard hospital doses and titration scales. The clinicians caring for the patient were blinded to the medication selected and the pump infusion rate. To ensure that the clinicians remained blinded, the CRI was labeled by the nurse caring for the patient as either “Drug A” or “Drug B”, and the CRI was formulated such that both medications were diluted, and the initial intravenous infusion rate was 1 mL/h. The CRI was then covered with a bag intended to prevent transmittance of ultraviolet light and to prevent clinicians from observing the subsequent rates if the CRI rate was altered. If NE was chosen, the CRI was started at a rate of 0.1 mcg/kg/min and could be titrated up by 0.1 mcg/kg/min every 15–30 min to a total dose of 0.5 mcg/kg/min if hypotension persisted. If DA was chosen, the CRI was started at a rate of 5 mcg/kg/min and could be titrated up by 2.5 mcg/kg/min every 15–30 min to a total dose of 15 mcg/kg/min if hypotension persisted. Once normotension was achieved (SBP > 90 mmHg or MAP > 70 mmHg), the frequency of SBP measurements and decision to increase or decrease the CRI rate was at the discretion of the attending veterinarian. If hypotension persisted when the predetermined maximum rate was reached for the initial vasopressor, vasopressin was added at a rate of 4 mU/kg/min. If hypotension persisted despite the administration of the first-line vasopressor and vasopressin, further vasopressor therapy could be initiated at the discretion of the attending clinician. If at any time the clinician asked to discontinue the study drug, the animal was removed from the study.

Blood pressure was measured via direct arterial blood pressure if available, or via Doppler, 811-B Doppler, Parks Medical Electronics Inc., Aloha, OR, USA, or an oscillometric device, Cardell 9402, Tampa, FL, USA, if direct pressure monitoring was not available. The choice of Doppler or an oscillometric device was at the discretion of the attending clinician. The Doppler blood pressure was recorded as SBP. All blood pressure measurements were performed by certified veterinary nurses, and measurements were taken in triplicate at all time points to ensure accuracy. Blood pressure measurements were scheduled every 15–30 min. All patients were placed on a continuous electrocardiographic (ECG) monitor, MDE Escort Prism SE, Invivo Research, Orlando, FL, USA.

Patient data collected included signalment, weight, survival, length of intensive care unit (ICU stay), time to normotension, need for a second vasopressor, occurrence of arrhythmias on the continuous ECG monitor, cardiovascular indices (heart rate [HR], SBP, MAP, shock index [SI]) at baseline and during vasopressor therapy, whether or not the vasopressor was weaned (based on clinician discretion), primary disease process and if applicable, source control surgery. Shock index was calculated as heart rate divided by systolic blood pressure. Successful weaning of vasopressors was defined as maintenance of normotension after discontinuation of the vasopressor medication, without a subsequent need to restart a vasopressor during hospitalization. Survival was recorded as survival to discharge, and death was recorded as either euthanasia or natural death.

The study design was approved by the Institutional Animal Care and Use Committee (#802776) and the Privately Owned Animal Protocol Review Committee (#246). All dogs were treated with intravenous fluids, early antibiotic administration, and gastrointestinal protectants, as is the standard of care at the study institution. The choice of specific medications, aside from the chosen vasopressor, was at the discretion of the attending veterinarian. To enable statistical analysis, a minimum of 10 dogs per treatment group were enrolled.

Continuous variables were assessed for normality using skewness and kurtosis tests for normality. Descriptive statistics are reported as mean +/− standard deviation (SD) for normally distributed variables and as median (range) for variables that are not normally distributed. The two-sample *t*-test or two-sample Wilcoxon rank-sum test was used to compare continuous variables between treatment groups depending on whether the variables were normally or not normally distributed, respectively. Categorical variables were described using counts or percents, and the Fisher’s exact test was used to compare these variables between treatment groups. The Wilcoxon signed-rank test was used to perform a series of baseline comparisons between (T0) and subsequent measurements (T1, T2, …, T10) of SBP, HR, and SI within each treatment group. In order to maximize sensitivity to find potentially important patterns and decrease the chance of a type I error, pairwise comparisons between early (defined as results from T1 to T5) and late (defined as results from T6 to T10) time periods were also performed using the Wilcoxon signed rank test. A *p*-value < 0.05 was considered significant for all evaluations. All statistical analyses were performed using a statistical software package, Stata 14.0 for Mac, Stata Corporation, College Station, TX, USA.

## 3. Results

Twenty-four dogs were initially enrolled. Two dogs were excluded, resulting in a total of twenty-two dogs included in the study. One dog was excluded because it was euthanized 30 min after enrollment, and the other was excluded because the CRI was started at an incorrect dose. Both excluded patients were in the NE group. Of the dogs that remained, ten dogs received NE as a first-line vasopressor, and twelve dogs received DA. There was no statistically significant difference in age, sex, weight, method of blood pressure measurement, respiratory rate, or baseline SBP, HR, or SI between the NE and DA groups at baseline (Table 1 and Supplementary Materials).

The majority of dogs were able to achieve normotension (17/22, 77.3%) during the study as measured by SBP. As only eight patients had MAP measurements available, but all had SBP measurements, statistical analysis was performed only on SBP measurements. There was no difference between the groups in the number of dogs that achieved normotension or the time required to attain normotension (Table 2). No dogs in the NE group were able to be weaned from the vasopressor, and four dogs in the DA group were able to be weaned, but there was no difference in the ability to wean the initial vasopressor between the groups (Table 2). There was no difference in the need for an additional vasopressor between the NE and DA groups. One patient in the DA group was treated with vasopressin, and the SBP increased from 80 mmHg to 127 mmHg. One patient in the NE group was normotensive, but decreased contractility was noted on an echocardiogram performed by a board-certified cardiologist. The patient was subsequently administered dobutamine to increase inotropy, and the SBP was similar before and after dobutamine. The patients that did not attain normotension and did not receive an additional vasopressor either died (*n* = 1, NE group) or were euthanized (*n* = 3, 2 in NE group and 1 in DA group) prior to reaching the maximum dose of the initial vasopressor.

In dogs treated with NE, median SBP at time zero was significantly lower than median SBP at time points 3–7 (Table 3). In dogs treated with DA, median SBP at time zero was also significantly lower than at all other time points except 3, 4, and 9 (Table 3). Median systolic blood pressure in the NE group was significantly higher than the DA group at measurement timepoints 1–5 (105 mmHg, range 20–140 vs. 90 mmHg, range 30–176, *p* = 0.0172), though not at timepoints 6–10 (117 mmHg, range 75–160 vs. 105 mmHg, range 40–171, 10 dogs, *p* = 0.16). When comparing timepoints 1–10, the NE group had a significantly higher median SBP compared to the DA group. The total number of patients decreased after T3 (NE group) and T2 (DA group) due to a positive response to the pressor and less frequent need for SBP measurements.

In dogs treated with NE, median HR at time zero (138, range 105–165) was not significantly higher than median HR measured at all other time points (Table 4). In dogs treated with DA, median HR at time zero (137, range 65–185) was also not significantly higher than median HR measured at all other time points (120, range 45–208, *p* = 0.35; however, HR decreased during timepoints 6–10 (98, range 56–150 bpm) compared to timepoints 1–5 (127, range 45–208, *p* = 0.021). The heart rate in the NE group was significantly higher than the DA group at measurement timepoints 6–10 (130 bpm, range 62–180 vs. 98 bpm, range 56–150, *p* = 0.023), though not at timepoints 1–5 (130 bpm, range 74–180 vs. 127, range 45–208, *p* = 0.69). When comparing timepoints 1–10, the NE group had a significantly higher HR compared to the DA group (130 bpm, range 62–180 vs. 120 bpm, range 45–208, *p* = 0.039).

In dogs treated with NE, median SI at time zero was significantly higher than median SI measured at time points 5, 6, and 7 (Table 5). In dogs treated with DA, median SI at time zero was also significantly higher than median SI measured at all other time points. There was no difference in SI between the NE and DA groups at measurement timepoints 1–5 (1.3, range 0.6–6.5 vs. 1.4, range 0.3–5, *p* = 0.36), or timepoints 6–10 (1.1, range 0.4–2.4 vs. 1.0, range 0.4–3.6, *p* = 0.69). When comparing timepoints 1–10, there was no difference in SI between the NE group and the DA group (1.2, range 0.4–6.5 vs. 1.2, range 0.3–5, *p* = 0.77).

T1–10 = 1st–10th measurements after starting NE or DA. There was no difference in the occurrence of arrhythmias (*p* = 1.0), with one patient in each group experiencing new-onset arrhythmias (Table 2). The dog in the NE group that developed arrhythmias had intermittent atrial premature complexes and intermittent sinus pauses with ventricular escape beats that were noted at a dose rate of 0.3 mcg/kg/min, and the dog remained on the NE CRI. The dog in the DA group that developed arrhythmias had an acute supraventricular tachycardia followed by a severe sinus bradycardia at a dose rate of 5 mcg/kg/min, and the DA CRI was discontinued. The arrhythmias resolved rapidly after discontinuation of the CRI and did not recur.

Underlying disease processes of dogs in both treatment groups are reported in Table 1. The overall mortality rate was 86.4%, and the median survival time was 2 days (IQR 1–4 days). Of the dogs that died, five died of their disease process, and 14 were euthanized. In the NE group, three dogs died of their disease process, and seven were euthanized. In the DA group, three dogs survived, two dogs died of their disease process, and seven were euthanized. There was no difference in survival between the NE and DA groups, both when evaluating patients that died of all causes (*p* = 0.320) or only those that died of their underlying disease process with euthanasia excluded (*p* = 0.221). The median length of ICU stay was 3 days (IQR 1–5.25), and there was no difference in length of ICU stay between the groups (*p* = 0.839).

## 4. Discussion

The majority of dogs in this study achieved normotension following vasopressor therapy, although there were no differences between the NE and DA groups in achievement of normotension, time to normotension, or the need for a second vasopressor. This is in contrast to a randomized controlled trial of NE versus DA for the treatment of septic shock in humans, which found that while only 31% of patients treated with DA achieved normotension, 93% of patients administered NE became normotensive; however, that study used a higher dose of norepinephrine than was used in this study [16]. It should be noted that the MAP goal in that study was 80 mmHg, whereas it was 70 mmHg in the current study. Additionally, targeting a higher MAP does not necessarily improve mortality, and the human Surviving Sepsis Campaign (SSC) guidelines recommend a target MAP of 65 mm Hg [22,23]. The optimal MAP target in vasodilatory shock is unclear, and human patients with septic shock randomized to either a target MAP of 65–70 mmHg or 80–85 mmHg did not result in a difference in 28-day or 90-day mortality. However, in human ICU patients with hypotension, it was found that the risk of acute kidney injury increased by 2% for each hour the MAP was below 70 mmHg [23].

It should be noted that normal blood pressure in dogs is variable, and an ideal blood pressure target for dogs in shock has yet to be determined [24]. Furthermore, most patients in the current study had Doppler blood pressure measurements, which are thought to correspond more closely to the SBP than the MAP in dogs, although the actual correlation is controversial and Doppler methods may overestimate SBP in hypotensive dogs [25,26]. Ideally, patients would have had dorsal pedal arterial catheters placed to measure direct arterial blood pressure; however, that was not logistically feasible in all patients in this study. As most of the patients had their blood pressure measured via Doppler, the statistical analysis focused on the SBP. Given that a normal MAP cannot be guaranteed by measurement of a normal SBP, particularly in patients with vasodilatory shock that may have low diastolic blood pressure, this is a limitation of the current study. It is possible that some patients were considered to have achieved normotension, based on SBP. remained hypotensive, and that a difference would be detectable if MAP was measured in all patients. Certified, experienced veterinary nurses took all non-invasive blood pressure measurements using the proper cuff size. This expertise and consistent technique are essential for accurate Doppler blood pressure measurements.

Although there was no difference in time to normotension between the NE and DA groups, there was a difference between baseline SBP and subsequent measurements in both groups, with an increase in blood pressure over time in both groups. The SBP in the NE group was higher than in the DA group at timepoints 1–5; however, this trend did not continue for subsequent timepoints. Due to patient dropout following death or euthanasia, only three patients remained in each group at time point 10. It may be that by this point, the study was too underpowered to detect a difference between NE and DA, and that in a larger group or one with fewer dropouts, NE would have maintained a higher SBP compared to DA.

Interestingly, the HR in the DA group at timepoints 1–5 was significantly higher than at timepoints 6–10. As dopamine has known beta-adrenergic effects at moderate doses (5–10 mcg/kg/min) it is expected to act as a positive chronotrope [27]. It is possible that the improvement in SBP may have resulted in a lessening of the hypotension-induced compensatory tachycardia; however, this would be expected in the NE group as well, and HR did not differ significantly from baseline in the NE group. In an experimental canine sepsis model, DA administration did not cause a change in HR in septic dogs, but did decrease HR in control dogs in a dose-dependent manner [8]. In that study, systemic vascular resistance also increased more in control dogs than in dogs in the sepsis group, so it may be that the beta-adrenergic effects of DA are diminished in patients with vasoplegia [8]. Dopamine has been reported to have variable effects on HR in anesthetized dogs, with HR decreasing at doses of 7 mcg/kg/min, and then increasing in a dose-dependent manner at higher doses [28]. Given that dopamine has variable effects on HR in anesthetized dogs, this may also hold true in non-anesthetized patients, and further investigation into the effect of moderate and high doses of dopamine on HR in dogs is warranted.

Shock index is the ratio of the HR to SBP, and a value of >0.8 has been associated with a need for vasopressors and increased rate of organ failure in human patients with sepsis [29]. In dogs presenting to an emergency service, an SI of >1.0 was associated with undifferentiated shock [30]. Shock index has also been shown to be associated with mortality in dogs that suffer from vehicular trauma, although admission SI and time to normalize SI were not associated with survival in dogs with hyperlactatemia and shock [31,32]. In this study, the baseline SI was significantly higher than most subsequent measurements, and the final SI measurement approached 1.0 in both groups. This suggests that both NE and DA were effective at improving SI, although the effects of therapeutics administered to patients outside of the study protocol (such as appropriate antibiotic therapy) cannot be evaluated. Despite the improvement in SI, the survival rate was low in this cohort of critically ill dogs. Further evaluation of the utility of SI in evaluating vasopressor responsiveness in dogs with and without vasodilatory shock in a larger canine population could prove useful.

There was no difference in survival between dogs that received NE or DA. In human patients, NE has been associated with a survival benefit in multiple studies and is the recommended first-line vasopressor for the treatment of septic shock [10,11,12,13,15,22]. Other analyses have failed to show a survival benefit for either vasopressor, and a 2016 Cochrane Review showed no difference in mortality between NE and DA in humans with undifferentiated shock [9,14]. The findings of the current study are consistent with the latter human studies. That said, the patient population in this study was small and had a high mortality rate. Of the 19 dogs that died, 14 were euthanized, which makes interpretation of the mortality data in this population difficult. It is possible that a survival benefit does exist, but this study was underpowered to detect such a benefit. A recent survey found that most board-certified veterinary criticalists follow the human guidelines and currently prescribe norepinephrine as a first-line vasopressor agent [33].

There was no difference in the occurrence of new-onset arrhythmias between the two groups, with only one dog in each group experiencing arrhythmias. This is in contrast to findings in human patients, where DA has been associated with increased risk of arrhythmias in multiple studies [9,10,12,14]. In the NE group, the dog that developed arrhythmias was noted to have intermittent atrial premature complexes and intermittent sinus pauses with ventricular escape beats that did not necessitate treatment or discontinuation of the CRI. In contrast, the dog in the DA group that experienced arrhythmias had an acute supraventricular tachycardia followed by a severe sinus bradycardia that necessitated rapid weaning of the medication, and the arrhythmia resolved once the DA was weaned. Although it is possible that these patients’ underlying illnesses contributed to the observed arrhythmias, the rapid resolution of arrhythmias in the patient receiving DA once the medication was weaned suggests that the medication played a role in arrhythmia generation. The patient that developed this arrhythmia was receiving treatment for lung abscessation and pneumonia and had no known underlying cardiac disease. It is possible that if a larger cohort were studied, a difference in the frequency or severity of arrhythmias could be detected.

Patients in this study were required to have at least a 15 mL/kg crystalloid bolus prior to enrollment. This volume of crystalloid is in contrast to the SSC’s recommendation to administer at least 30 mL/kg IV crystalloid during resuscitation [17]. Although it is a strong recommendation by SSC, it is considered to have a low quality of evidence to support it [34]. The crystalloid volume of 30 mL/kg was chosen based on a retrospective study that found a correlation between mortality and fluid volume administered, with patients receiving between 15–45 mL/kg crystalloid having the lowest mortality [34,35]. In determining inclusion criteria for this study, we felt that some amount of fluids had to be given prior to enrollment to reduce the risk that hypovolemia was the cause of hypotension in enrolled patients. However, in anticipation of patients having individual fluid requirements based their underlying disease process, a lower volume of crystalloid was chosen as part of the inclusion criteria so that clinicians would not be required to give a minimum of 30 mL/kg in crystalloid if the patient did not appear to be volume responsive or there was concern for volume overload, but clinicians could give more than 15 mL/kg if indicated. Ultimately, the inclusion criteria used meant that vasodilatory shock was defined as hypotension refractory to IV fluid administration, rather than based on clinical signs of vasodilation such as injected mucous membranes, a rapid capillary refill time, or hyperkinetic pulses. We did not include these clinical signs as inclusion criteria since mucous membrane color and pulse quality are inherently subjective, and capillary refill time measurement has been shown to have poor interobserver agreement [36,37]. It is possible that, because of our inclusion criteria, some patients may have had concurrent hypovolemia when they were started on either NE or DA.

The primary limitation of this study is its small sample size, which could have limited our ability to detect differences between dogs that received NE or DA. Another limitation is that although we attempted to measure BP and titrate the initial vasopressor every 15–30 min, the intervals between measurements varied considerably due to changes in patient load and staff availability. We also chose our starting dose and titration doses of NE and DA based on our standard hospital protocol; however, there are varied reported doses of NE and DA, and in some patients, it would be reasonable to titrate a vasopressor more or less quickly. Although the dose range for NE can be as high as 2 mcg/kg/min, we chose a lower dose at which vasopressin could be administered. This is consistent with the SCC guidelines that recommend a threshold dose of NE of 0.25–0.5 mcg/kg/min before starting vasopressin [17]. In order to partially compensate for the low number of animals included, groupings were made. However, it should be noted that this approach may homogenize changes occurring at different grouped times. In addition, the loss to follow-up over time led to unbalanced comparisons between groups, which could have biased the results. Due to a lack of guidelines for when to start a second vasopressor when patients are receiving DA, we used available pharmacologic data to estimate the dose at which the alpha-adrenergic effects of dopamine would be in effect, but this is likely variable in individual patients [6]. Additionally, we did not include timing or dosing of corticosteroids in our study protocol. As corticosteroids can restore the sensitivity of the vasculature to alpha-adrenergic agonists, they remain an important aspect of the management of patients in refractory vasodilatory shock. [38] Future studies that include corticosteroids in the study protocol would be beneficial. Lastly, aside from the study medication, other treatments administered were not standardized, which also may have influenced the outcome. Future studies with a larger patient cohort facilitated by multicenter collaboration, as well as staff dedicated solely to data acquisition, could help to detect differences that may exist.

## 5. Conclusions

In conclusion, this is the first evaluation of NE versus DA in dogs with naturally occurring vasodilatory shock. The majority of dogs achieved normotension, and there was no difference between the NE and DA groups in the achievement of normotension. Although patients in the NE group initially demonstrated higher SBP than in the DA group, this did not hold true at subsequent timepoints. Future prospective studies evaluating the ideal first-choice vasopressor for the treatment of hypotension in dogs with vasoplegic shock are indicated.

## Figures and Tables

**Table 1:** Baseline characteristics of dogs with vasodilatory shock randomized to receive norepinephrine or dopamine as a first-line vasopressor.

	Norepinephrine (*n* = 10)	Dopamine (*n* = 12)	*p* Value
Age (years) mean ± SD	7.69 ± 4.22	8.15 ± 5.31	0.779
Sex	5 female spayed 1 female intact 3 male castrated 1 male intact	3 female spayed 1 female intact 8 male castrated	0.316
Weight (kg) mean ± SD	25.1 ± 5.26	20.0 ± 6.08	0.194
Underlying disease process	Pneumonia (4) Intestinal perforation (2) Surgical site infection (1) Septic lymphadenitis (1) Infected mammary mass (1) Bite wounds (1) Parvovirus (1) Vehicular trauma (1)	Pneumonia (4) Pancreatitis (2) Hepatic abscess (1) Intestinal perforation (1) Histoplasmosis (1) Hypoadrenocorticism, urinary tract infection (1) Acute kidney injury (1) Post-cardiopulmonary arrest, acute respiratory distress syndrome (1) Undifferentiated shock (1)	N/A
Respiratory rate (breaths/minute) mean ± SD	26.2 ± 6.96	32.3 ± 15.0	0.127
Systolic blood pressure (mmHg) median (IQR)	85 (45, 103)	65.5 (40, 116)	0.11
Heart rate (bpm) median (IQR)	138 (105, 165)	137 (65, 185)	0.47
Shock index median (IQR)	1.6 (1.1, 2.9)	1.85 (0.6, 3.3)	0.62
Method of blood pressure measurement	6 Doppler 1 oscillometric 3 direct arterial	8 Doppler 1 oscillometric 3 direct arterial	1.00

SD = standard deviation, IQR = interquartile range. Two patients in the NE group diagnosed with pneumonia had concurrent infections (1 each with septic lymphadenitis and bite wounds). One patient in the DA group was diagnosed with concurrent pneumonia and an intestinal perforation.

**Table 2:** Response to vasopressor administration in dogs with vasodilatory shock randomized to receive norepinephrine or dopamine as a first-line vasopressor.

	Norepinephrine (*n* = 10)	Dopamine (*n* = 12)	*p* Value
Number of dogs that achieved normotension	7	10	0.62
Time required to attain normotension (hours) mean ± SD	1.29 ± 1.24	1.43 ± 0.89	0.80
Ability to wean vasopressor	0	4	0.096
Dogs requiring an additional vasopressor	1	2	1.0
Number of dogs with new arrhythmias	1	1	1.0

SD = standard deviation.

**Table 3:** Systolic blood pressure (mmHg) at sequential time points in dogs with vasodilatory shock randomized to receive norepinephrine or dopamine as a first-line vasopressor.

	T0	T1	T2	T3	T4	T5	T6	T7	T8	T9	T10
NE median (IQR)	85 (74, 96)	101 (78, 108)	96 (88, 111)	**107 (96, 121)**	**110 (96, 127)**	**120 (103, 130)**	**125 (115, 131)**	**113 (106, 128)**	114 (106, 140)	109 (43, 119)	117 (104, 120)
*p* value	N/A	0.293	0.215	**0.022**	**0.031**	**0.016**	**0.031**	**0.031**	ND	ND	ND
N	10	10	10	10	9	7	6	6	5	5	3
DA median (IQR)	65.5 (54, 92)	**92.5 (71, 121)**	**91.5 (74, 118)**	87.5 (63, 94)	86.5 (69, 99)	**103 (73, 133)**	**100 (85, 136)**	**110 (100, 128)**	**93 (80, 132)**	95 (72, 150)	**120 (91, 141) 113 ± 34**
*p* value	N/A	**0.002**	**0.001**	0.148	0.125	**0.006**	**0.02**	**0.031**	**0.031**	0.063	**0.031**
n	12	12	12	10	10	10	9	7	6	6	6

IQR = interquartile range, ND = not done since <6 patients in group. *p* value = difference between baseline (T0) and subsequent measurements (T1, T2….). T1–10 = 1st–10th measurements after starting NE or DA. Bold values indicate measurements and *p* values that are statistically different from baseline (T0).

**Table 4:** Heart rate (bpm) at sequential time points in dogs with vasodilatory shock randomized to receive norepinephrine or dopamine as a first-line vasopressor.

	T0	T1	T2	T3	T4	T5	T6	T7	T8	T9	T10
NE median (IQR)	138 (107, 161)	133 (112, 155)	138 (105, 156)	128 (104, 150)	138 (98, 145)	118 (89, 141)	137 (96, 152)	117 (91, 154)	130 (93, 167)	121 (97, 167)	1111 (82, 152)
*p* value	N/A	0.383	0.21	0.121	0.102	0.063	>0.999	0.438	ND	ND	ND
n	10	10	10	10	9	7	6	6	5	5	3
DA median (IQR)	137 (97, 150)	128 (97, 148)	121 (89, 150)	128 (117, 149)	135 (97, 146)	118 (87, 147)	**115 (76, 135)**	**114 (90, 124)**	**94 (73, 115)**	106 (89, 128)	94 (85, 143)
*p* value	N/A	0.43	0.691	0.77	0.445	0.065	**0.023**	**0.047**	**0.031**	0.156	0.094
n	12	12	12	10	10	10	9	7	6	6	6

IQR = interquartile range, ND = not done since <6 patients in group. *p* value = difference between baseline (T0) and subsequent measurements (T1, T2….). T1–10 = 1st–10th measurements after starting NE or DA. Bold values indicate measurements and *p* values that are statistically different from baseline (T0).

**Table 5:** Shock index at sequential time points in dogs with vasodilatory shock randomized to receive norepinephrine or dopamine as a first-line vasopressor.

	T0	T1	T2	T3	T4	T5	T6	T7	T8	T9	T10
NE mean ± SD	1.75 ± 0.56	1.79 ± 1.3	1.83 ± 1.7	1.72 ± 1.7	1.69 ± 1.7	**1.03 ± 0.34**	**1.03 ± 0.31**	**1.05 ± 0.44**	1.14 ± 0.48	1.24 ± 0.68	1.03 ± 0.42
*p* value	N/A	0.415	0.126	0.074	0.150	**0.018**	**0.046**	**0.035**	ND	ND	ND
n	10	10	10	10	9	7	6	6	5	5	3
DA mean ± SD	1.93 ± 0.77	**1.51 ± 0.80**	**1.49 ± 0.73**	**1.58 ± 0.61**	1.76 ± 1.3	**1.34 ± 0.82**	**1.24 ± 0.94**	**1.24 ± 0.81**	**0.97 ± 0.36**	1.18 ± 0.48	**1.02 ± 0.50**
*p* value	N/A	**0.017**	**0.005**	**0.019**	0.138	**0.005**	**0.015**	**0.018**	**0.028**	0.074	**0.028**
n	12	12	12	10	10	10	9	7	6	6	6

SD = standard deviation, *p* value = difference between baseline (T0) and subsequent measurements (T1, T2….), ND = not done since <6 patients in group. Bold values indicate measurements and *p* values that are statistically different from baseline (T0).

## Data Availability

The data for this study can be found at: https://docs.google.com/spreadsheets/d/1V3w-mews-75kBWzBapMGOpaNQYTM_e5PShOMC_IOzMw/edit?usp=sharing (accessed on 28 June 2025).

## Supplementary Materials

The following supporting information can be downloaded at: https://www.mdpi.com/article/10.3390/vetsci12090832/s1.

## Abbreviations

The following abbreviations are used in this manuscript:

NE	norepinephrine
DA	dopamine
CRI	constant rate infusion
SBP	systolic blood pressure
MAP	mean arterial blood pressure
HR	heart rate
SI	shock index
